# The Impact of Mask-Wearing on Allergic Rhinitis Symptoms During the COVID-19 Pandemic Among the Saudi Population: A Cross-Sectional Study

**DOI:** 10.7759/cureus.59937

**Published:** 2024-05-08

**Authors:** Baha Eldien Atta, Tahani F Alanazi, Khalid Atallah M Alsayahani, Nadyah K Al Najar, Ghayda M Alyamani, Omar A Aljasser, Lama Ahmad, Raghad Aljohani, Ghadeer A Al Bensaad

**Affiliations:** 1 College of Medicine, Department of ENT, Majmaah University, Al Majma'ah, SAU; 2 Faculty of Medicine, University of Tabuk, Tabuk, SAU; 3 College of Medicine, Majmaah University, Al Majma'ah, SAU; 4 College of Medicine, Almaarefa University, Riyadh, SAU; 5 College of Medicine, King Saud bin Abdulaziz University for Health Sciences (KSAU-HS), Jeddah, SAU; 6 Faculty of Medicine, Taif University, Taif, SAU; 7 Faculty of Medicine, Department of Dermatology, University of Tabuk, Tabuk, SAU; 8 Medicine and Surgery, King Faisal University, Al Hofuf, SAU

**Keywords:** allergens, allergic rhinitis, covid-19, face mask, saudi arabia

## Abstract

Background: Allergic rhinitis (AR) is an inflammatory disease of the nasal mucosa caused by certain allergens that may be found indoors or outdoors, and it greatly impacts the patient’s quality of life. The COVID-19 epidemic offers an excellent chance to examine how using a face mask affects allergy.

Aim: The present study aimed to evaluate the impact of face mask wearing on AR symptoms among subjects living in the northern, southern, eastern, western, and central regions of Saudi Arabia.

Subjects and methods: This cross-sectional, survey-based study was undertaken in all Saudi Arabia regions in 2022. We included female and male adults living in Saudi Arabia who have AR and completed the Arabic version of an electronic self-administered questionnaire.

Results: The overall received responses were 2252. According to the study eligibility criteria, we assessed the data of 470 participants who self-reported to have been diagnosed with AR. There was no significant change in the proportions of nasal symptoms severity before and after wearing face masks during the pandemic (p = 0.867), while a significant negative change was observed in the rates of moderate and severe ophthalmic symptoms (p < 0.001). The need for AR drugs was significantly increased during the pandemic (no need for drugs was reported by 45.3% before the pandemic and by 37.9% during the pandemic, p < 0.001). However, the use of AR drugs was significantly associated with the improvement of AR symptoms (p < 0.001); complete and partial eliminations of AR symptoms were higher with the use of masks during the pandemic (11.3% and 36.8%) than before the pandemic period (10.6% and 34.5%).

Conclusions: Face mask usage was not associated with improved symptoms or severity of AR. Wearing the masks was associated with increased severity of ophthalmic symptoms. The use of face masks was associated with a significant increase in the partial and complete elimination of AR symptoms with the use of AR drugs, particularly with the constant use of masks.

## Introduction

Allergic rhinitis (AR) is an inflammatory disease of the nasal mucosa caused by certain allergens that may be found indoors or outdoors. On exposure to these allergens, immunoglobulin E activates the mast cells or basophils present in the nasal mucosa, with subsequent degranulation and release of vasoactive mediators, such as histamine that initiates the inflammation [1].

AR is primarily manifested by nasal symptoms, such as congestion, discharge, itching, and sneezing, as well as ocular symptoms, like itching, redness, and tearing [2]. The impact of AR on patients is substantial; it adversely affects their social life, academic performance, work productivity, and sleep quality [3].

Globally, the prevalence of AR in adults ranges from 10% to 30%, while in children, it is about 40% [4]. A study done in the Middle East reported a prevalence of AR symptoms among participants in Western Saudi Arabia at 24% [5]. Another study reported that the prevalence of intermittent AR in Saudi Arabia was 65% compared to the persistent form of AR (35%) [6]. Saudi Arabia is well-known for its repeated sandstorms throughout the year. These storms carry a variety of microorganisms and dust particles that can trigger or aggravate respiratory diseases, such as AR and asthma [7].

Concerning the management of AR, avoidance of allergens and environmental controls are considered the first-line treatment of AR [8]. Face mask usage during pollen seasons has been recommended as an avoidance method [9]. Reducing exposure to allergens increases the efficacy of medical treatment, thereby improving the quality of life. However, the role of allergen control is debatable because the full implementation of environmental controls and protective methods is difficult. In addition, it is not based on the best-quality evidence [10].

Due to the COVID-19 pandemic, people have continued to wear masks outdoors and non-residential indoors. A significant decrease in the incidence of AR in 2020 (16.3%) than in 2019 (19.5%) has been reported [11]. Therefore, the pandemic provides a good opportunity to assess the effect of face mask usage on allergy symptoms. 

The current study aimed to evaluate the impact of face mask wearing on AR symptoms among subjects living in different regions of Saudi Arabia.

## Materials and methods

Nature of the study

This was a cross-sectional, survey-based study. The study was conducted between January and October 2022. It was undertaken among adult patients with AR who live in any region of Saudi Arabia.

Inclusion and exclusion criteria

We included both female and male adults living in Saudi Arabia who have AR and who completed the questionnaire. We excluded participants who did not have AR symptoms, were younger than 18 years old, cannot read or write, or were living outside Saudi Arabia.

Sample size and sampling technique

The sample size was calculated using the formula n = z2pq\d 2. According to the most conservative scenario of a 50% prevalence of AR, with a 5% margin of error, and a 95% confidence interval, the minimum required sample size was 384. After keeping the non-response rate at 10%, the required total sample size was 430 subjects. The participants were randomly selected by a simple random sampling technique.

Data collection

The participants were requested to complete an Arabic version of an electronic self-administrated questionnaire. The questionnaire has been previously developed by a team experienced in allergy [12]. To attain the purpose of this study, the questionnaire was translated into Arabic. The first and second sections of the questionnaire included sociodemographic data of the participants and their medical history involving allergic symptoms during the pre-pandemic period. The third section included inquiries about using face masks as part of their COVID-19 defenses and the kind of masks. Then, the participants were asked to rate the overall nasal and ophthalmic symptoms of AR, the most worrying AR symptoms, the need for medical attention, and the effectiveness of AR drugs before the pandemic and with mask-wearing during the pandemic.

Ethical considerations

The study obtained ethical approval from the Research Ethics Committee of Majmaah University, Saudi Arabia (ethical number: MUREC-Oct.6/COM-2022/14-3).

The survey included an explanatory letter to show the study objectives, methodology, risks, and benefits. Subjects who agreed to fill out the questionnaire indicated that they agreed to participate in the study. The participants’ confidentiality was preserved by keeping the data anonymous.

Statistical analysis

All tables and statistical analyses were performed using the IBM SPSS Statistics for Windows, version 22.0 (released 2013, IBM Corp., Armonk, NY). Categorical variables were presented as numbers and percentages. We used the McNemar and marginal homogeneity tests for comparing the paired responses of the participants (before and during the pandemic). For all analyses, a p-value < 0.05 was considered statistically significant.

## Results

The overall received responses were 2252. According to the study eligibility criteria, we assessed the data of 470 participants who self-reported having been diagnosed with AR. The highest percentages (94.0%) were Saudi. Females constituted 339 (72.1%) of the participants and 131 (27.9%) were males. The most frequent age groups were 18-24 and 25-34 years (42.3% and 21.9%, respectively). Single (49.6%) and married (49.6%) subjects were the most frequent. Most of the participants have a bachelor’s degree (68.7%) and were students (37.0%) or employees (30.0%). All regions of Saudi Arabia were presented; however, the western (36.8%) and central (22.1%) regions were the most common (Table 1).

**Table 1:** Sociodemographic characteristics of the study participants having allergic rhinitis (N = 470)

		N	%
Sex	Females	339	72.1%
	Males	131	27.9%
Age, years	18-24	199	42.3%
	25-34	103	21.9%
	35-44	95	20.2%
	>45	73	15.5%
Nationality	Saudi	442	94.0%
	Non-Saudi	28	6.0%
Marital status	Widowed	7	1.5%
	Single	233	49.6%
	Married	214	45.5%
	Divorced	16	3.4%
Academic level	Elementary school	3	0.6%
	High school	105	22.3%
	Bachelor’s degree	323	68.7%
	Master’s degree	26	5.5%
	Intermediate school	13	2.8%
Employment status	Student	174	37.0%
	Self-employed	36	7.7%
	Not working	119	25.3%
	Employee	141	30.0%
Region	Southern	67	14.3%
	Eastern	74	15.7%
	Northern	52	11.1%
	Western	173	36.8%
	Central	104	22.1%

Table 2 shows the medical history of the study participants having AR. They reported nasal allergy symptoms, such as sneezing and congestion (83.8%), watery rhinorrhea (73.2%), itching (71.1%), and hyposmia (42.8%). Itching (53.2%), redness (44.9%), and burning (40.6%) were the most frequent ophthalmic symptoms. Otologic symptoms were less frequent, mainly in the form of middle ear effusion (11.5%) and eustachian tube dysfunction (10.6%). Cough and dryness of the larynx were the most frequent laryngeal symptoms (70.9% and 53%, respectively). Palatal itching and frontal or periorbital headaches constituted 53.0% and 68.9%, respectively. Furthermore, fatigue was highly reported (63.6%) symptoms by the study participants. All the reported allergy symptoms increased during winter (46.4%) and autumn (24.9%). More than half (51.3%) were sometimes taking medications for relieving symptoms of allergy. Approximately one-third (34.3%) documented a history of other allergies, and more than one-half (51.9%) had a family history of allergic diseases, such as allergies, asthma, atopic dermatitis, or eczema. 

**Table 2:** Medical history of the study participants having allergic rhinitis (N = 470)

	N	%
Nasal symptoms		
Sneezing, congestion	394	83.8%
Watery rhinorrhea	344	73.2%
Itching	334	71.1%
Hyposmia	201	42.8%
Ophthalmic symptoms		
Redness	211	44.9%
Itching	250	53.2%
Conjunctivitis	73	15.5%
Burning	191	40.6%
Otologic symptoms		
Middle ear effusion	54	11.5%
Eustachian tube dysfunction	50	10.6%
Laryngeal symptoms		
Scratchiness	177	37.7%
Dry	249	53.0%
Irritated	177	37.7%
Cough	333	70.9%
Oral symptoms		
Palatal itching	249	53.0%
Hypogeusia	153	32.6%
Facial symptoms		
Frontal or periorbital headaches	324	68.9%
Other symptoms		
Food hypersensitivity	93	19.8%
Fatigue	299	63.6%
When do these symptoms increase?		
During Summer	75	16.0%
During Winter	218	46.4%
During Spring	60	12.8%
During Autumn	117	24.9%
Did you take medication to reduce the symptoms?		
Always	110	23.4%
Usually	82	17.4%
Sometimes	241	51.3%
Never	37	7.9%
A history of other allergies	161	34.3%
Family history of allergic diseases	244	51.9%

During the pandemic, 287 (61.1%) of the study participants worked from the office regularly, 65 (13.8%) worked from home, and 118 (25.1%) did not work. More than half (54.7%) of the study participants stated that they wore the face mask if necessary, and 30.6% constantly wore face masks outside of their homes and workplaces, while 14.7% rarely used the mask.

The study participants scored their AR symptom severity before and after wearing face masks. There was no significant association between the use of masks during the pandemic and the reported rates of the most disturbing allergic symptoms (p = 0.234). Furthermore, there was no significant change in the proportions of nasal symptoms severity before and after wearing face masks during the pandemic (p = 0.867), while a significant change was observed in the rates of ophthalmic symptoms (p < 0.001). With the mask used during the pandemic, the proportions of subjects who reported severe (8.3%) and moderate (23.0%) ophthalmic symptoms significantly increased, while the proportions of those who reported mild (24.5%) or no symptoms (44.3%) significantly decreased. Furthermore, the requirement for medical attention for allergy symptoms did not show a significant difference with mask use during the pandemic (p = 0.865). The need for AR drugs was significantly increased during the pandemic (no need before and during the pandemic were 45.3% and 37.9%, respectively); however, the complete and partial elimination of symptoms (11.3% and 36.8%) was significantly increased with the use of masks during the pandemic than in the pre-pandemic period (19.6% and 34.5%, p<0.001) (Table 3).

**Table 3:** Frequency of allergic rhinitis symptoms before and during the COVID-19 pandemic

		Before the pandemic (no mask)		During the pandemic (using a mask)		P-value
		N	%	N	%	
How would you characterize your allergic nasal symptoms?	Severe	32	6.8%	72	15.3%	0.867
	Moderate	205	43.6%	179	38.1%	
	Mild	162	34.5%	116	24.7%	
	No	71	15.1%	103	21.9%	
How would you characterize your allergic ophthalmic symptoms?	Severe	19	4.0%	39	8.3%	<0.001*
	Moderate	75	16.0%	108	23.0%	
	Mild	160	34.0%	115	24.5%	
	No	216	46.0%	208	44.3%	
Did you require medical attention for your allergy symptoms?	No	305	64.9%	302	64.3%	0.865
	Yes	165	35.1%	168	35.7%	
How did your allergic rhinitis drugs alter your symptoms?	Eliminate	50	10.6%	53	11.3%	<0.001*
	Partially eliminate	162	34.5%	173	36.8%	
	No benefit	45	9.6%	66	14.0%	
	No need	213	45.3%	178	37.9%	
What allergic symptoms worried you the most?	Nasal congestion	161	34.3%	141	30.0%	0.234
	Nasal discharge	97	20.6%	90	19.1%	
	Nasal itching	79	16.8%	102	21.7%	
	Sneezing	133	28.3%	137	29.1%	

Table 4 shows an absence of significant associations between the type of mask and the scored overall severity of AR symptoms during the pandemic period (all p values > 0.05).

**Table 4:** Associations between the type of mask and the allergic rhinitis symptoms

		Type of mask						P-value
		Fabric		Surgical		N95		
		N	%	N	%	N	%	
How would you characterize your allergic nasal symptoms?	Severe	62	15.3%	6	13.6%	4	19.0%	0.420
	Moderate	154	38.0%	20	45.5%	5	23.8%	
	Mild	98	24.2%	13	29.5%	5	23.8%	
	No	91	22.5%	5	11.4%	7	33.3%	
How would you characterize your allergic ophthalmic symptoms?	Severe	35	8.6%	2	4.5%	2	9.5%	0.574
	Moderate	94	23.2%	10	22.7%	4	19.0%	
	Mild	93	23.0%	16	36.4%	6	28.6%	
	No	183	45.2%	16	36.4%	9	42.9%	
Did you require medical attention for your allergy symptoms?	No	265	65.4%	23	52.3%	14	66.7%	0.218
	Yes	140	34.6%	21	47.7%	7	33.3%	
How did your allergic rhinitis drugs alter your symptoms?	Eliminate	43	10.6%	7	15.9%	3	14.3%	0.732
	Partially eliminate	153	37.8%	14	31.8%	6	28.6%	
	No need	150	37.0%	19	43.2%	9	42.9%	
	No benefit	59	14.6%	4	9.1%	3	14.3%	
What allergic symptoms worried you the most?	Nasal congestion	114	28.1%	20	45.5%	7	33.3%	0.282
	Nasal discharge	80	19.8%	6	13.6%	4	19.0%	
	Nasal itching	92	22.7%	5	11.4%	5	23.8%	
	Sneezing	119	29.4%	13	29.5%	5	23.8%	

Table 5 shows a significant improvement in allergic ophthalmic symptoms with the rare use (4.3%) of the mask than the constant (6.3%) or the infrequent “when necessary” use of the mask (10.5%). In addition, there was a significant association between the rare use of the mask and the increased reporting of the absence of allergic ophthalmic symptoms during the pandemic (p = 0.004). However, the perceived altering effect of AR drugs was significantly associated with the constant use of the masks (p = 0.001). The proportions of respondents who reported complete (14.6%) or partial (41.0%) relief with the use of AR drugs were significantly higher among constant mask users than their counterparts.

**Table 5:** Associations between the regularity of face mask use and allergic rhinitis symptoms

		Do you employ face masks as part of your COVID-19 defenses?						
		If necessary		Always		Rarely		
		N	%	N	%	N	%	P-Value
How would you characterize your allergic nasal symptoms?	Severe	32	12.5%	28	19.4%	12	17.4%	0.101
	Moderate	96	37.4%	60	41.7%	23	33.3%	
	Mild	73	28.4%	30	20.8%	13	18.8%	
	No	56	21.8%	26	18.1%	21	30.4%	
How would you characterize your allergic ophthalmic symptoms?	Severe	27	10.5%	9	6.3%	3	4.3%	0.004*
	Moderate	52	20.2%	40	27.8%	16	23.2%	
	Mild	68	26.5%	40	27.8%	7	10.1%	
	No	110	42.8%	55	38.2%	43	62.3%	
Did you require medical attention for your allergy symptoms?	No	164	63.8%	89	61.8%	49	71.0%	0.413
	Yes	93	36.2%	55	38.2%	20	29.0%	
How did your allergic rhinitis drugs alter your symptoms?	Eliminate	25	9.7%	21	14.6%	7	10.1%	0.001*
	Partially eliminate	97	37.7%	59	41.0%	17	24.6%	
	No need	102	39.7%	52	36.1%	24	34.8%	
	No benefit	33	12.8%	12	8.3%	21	30.4%	
What allergic symptoms worried you the most?	Nasal congestion	86	33.5%	36	25.0%	19	27.5%	0.090
	Nasal discharge	57	22.2%	25	17.4%	8	11.6%	
	Nasal itching	47	18.3%	36	25.0%	19	27.5%	
	Sneezing	67	26.1%	47	32.6%	23	33.3%	

## Discussion

AR has a great impact on the patient’s quality of life, and it is substantial to be controlled and provided the best treatment [13]. The COVID-19 epidemic offers an excellent chance to examine how using a face mask affects allergy. The rationale for using the face mask during the COVID-19 pandemic is reducing the transmission of infection by the severe acute respiratory syndrome coronavirus by preventing pathogen penetration. Face masks can potentially lower the burden of other inhaled airborne particles, including allergens and air pollutants [14]. Therefore, the present study aimed to evaluate the impact of face mask-wearing on AR symptoms among subjects living in different regions of Saudi Arabia.

This study showed that face masks were not effective in improving the symptoms or severity of AR. There was no significant association between the severity of the self-reported nasal symptoms of AR and the use of face masks whatever the type of mask used. Moreover, the rates of the most disturbing allergic symptoms did not significantly differ with the mask used during the pandemic. Surprisingly, we observed a significant increase in the severity of ophthalmic symptoms with mask use, particularly with constant use of masks. However, the use of masks significantly increased the efficacy of medical treatment. Although the need for AR drugs was significantly increased during the pandemic, the rates of complete and partial improvement were significantly increased. Moreover, the perceived altering effect of AR drugs was significantly associated with the constant use of the masks.

The findings in the present study disagree with the findings of Mengi et al. [12] who reported a statistically significant decrease in both nasal and ocular symptoms of patients after constant mask use outside homes and workplaces during the COVID-19 pandemic. This contradiction might be attributed to the different criteria of the recruited participants as the authors included only patients with isolated pollen allergy who were identified by the skin-prick test and who were symptomatic during the study period, while they excluded those who were sensitive to indoor allergens, such as house dust mites, fungal spores, animal epithelial materials, and cockroaches. It has been reported that the sizes of different types of pollens (10 to 100 μm) can be filtered by the standard surgical, which filters particles larger than 3 μm. However, some fungal spores are of 2 μm size, which is too small to be filtered by the surgical masks and can be only filtered by the N95 masks, which filter particles as small as 0.04 μm [15-16].

The observed lack of protective effects of mask-wearing on nasal symptoms of AR in this study is potentially attributable to allergens that are not removed by face mask filtration due to unfiltered airflow through either defective mask seal edges or allergen exposure when not wearing the mask. These allergens could initiate allergic responses under face mask-wearing conditions [17]. Despite the insignificant impact of mask-wearing on the severity of nasal symptoms, we unexpectedly observed a significant increase in the severity of ophthalmic symptoms with mask use, particularly with constant use of the mask. This is not in line with the study of Dror et al. [17] who concluded that the use of face masks during the COVID-19 pandemic reduced allergic nasal symptoms but did not affect ophthalmic symptoms in nurses with AR symptoms. This observation highlights the possible role of face masks in lowering the exposure of the upper airways to allergens; nonetheless, the eye's conjunctiva remains unprotected and exposed to irritating allergens. On the other hand, the exacerbation of the severity of ophthalmic symptoms with the use of the masks might result from the continued stimulation of the parasympathetic afferents in the nasal cavity by the inspired allergens, which initiate the nasal ocular reflex and affect the eyes [18]. Another study reported a significant decrease in both nasal and ophthalmic symptoms with mask-wearing during the pandemic among 14 subjects having grass pollen allergy [19]. There is limited literature on the efficacy of face masks on the symptoms and severity of AR. Some randomized clinical trials evaluated the efficacy of nasal filters, and they reported significant improvement of nasal symptoms in patients under repeated exposure in an environmental exposure unit [20], in a natural setting, and during a pollen season [21].

The pharmacologic treatment of AR constitutes a significant economic burden. There is a continued increase in the annual cost of antihistamines and intranasal corticosteroids [22].

Fortunately, this study revealed a significant increase in the efficacy of antiallergic drugs with mask use, with an observed increase in the reported rates of complete and partial improvement. Considering the more reasonable costs of the masks in comparison to the medical treatment, this finding might have a positive impact on the costs of treatment and encourages the wearing of masks by AR patients.

Limitations

This study has some limitations. The effects of COVID-19 preventive measures other than face mask usage were not taken into consideration in this study. Lockdown and spending less time outdoors as recommended by health authorities might have reduced the exposure of the participants to allergens. The diagnosis of AR was based on self-reporting and was not confirmed by specific investigations, such as skin prick and blood tests. In addition, online surveys may be subject to a misunderstanding of some operational definitions.

## Conclusions

Face mask usage was not associated with improved symptoms or severity of AR. Wearing masks was associated with increased severity of ophthalmic symptoms. The use of face masks was associated with a significant increase in partial and complete elimination of AR symptoms with the use of AR drugs, particularly with the constant use of masks.
